# Gamma-delta T-cell acute lymphoblastic lymphoma/leukemia: a report of a rare entity

**DOI:** 10.1007/s12308-024-00578-7

**Published:** 2024-03-25

**Authors:** Giby V. George, Maggie Kajstura, Andrew G. Evans, Chauncey R. Syposs

**Affiliations:** Department of Pathology and Laboratory Medicine, University of Rochester Medical Center, Rochester, NY 14642 USA

**Keywords:** T-cell leukemia, Gamma-delta, T-cell lymphoma, T-cell acute lymphoblastic leukemia

## Abstract

Gamma delta (γδ) T-cell acute lymphoblastic leukemia/lymphoma (T-ALL) is a rare, aggressive subtype of T-lymphoid leukemia that accounts for only 9–12% of all T-ALL cases. Herein, we report the case of an 8-year-old boy who presented with facial swelling, shortness of breath, and progressive cervical and axillary lymphadenopathy. Pathological examination, flow cytometry (Navios, Beckman Coulter ClearLLab 10C 10-color T-cell panel [containing FITC-labeled TCR γδ antibody]), chromosomal analysis, interphase FISH, and targeted DNA-based NGS (34-gene Illumina TruSeq Myeloid Panel) were performed. Flow cytometry evaluation of a lymph node biopsy specimen revealed an immature T-cell population positive for CD4, CD3, CD2 (subset positive), CD5, CD7, CD38, CD1a, cytoplasmic terminal deoxynucleotidyl transferase (cyto-TdT), CD30 (subset positive), and T-cell receptor (TCR) gamma delta (γδ). Microscopic examination of an enlarged lymph node and bone marrow showed involvement by a dense, diffuse, neoplastic infiltrate. Interphase FISH revealed a copy number loss of *PDGFRB* (5q32) in 90.5% of interphase nuclei. Targeted DNA-based NGS detected a tier II oncogenic variant in *NOTCH1* (c.7375C > T, p.Gln2459Ter) at a VAF of 21%. This case of γδ T-ALL highlights a rare entity and adds to the literature, albeit scant, which may aid in better recognition and classification.

## Introduction

Gamma delta T-cell acute lymphoblastic leukemia ((γδ) T-ALL) is an aggressive subtype of T-lymphoid leukemia [1]. The differential diagnosis in most cases includes other acute leukemias. Since γδ T-ALL is rare and accounts for only 9–12% of all T-ALL cases, additional studies are needed to better aid in the classification and understanding of disease biology [2, 3]. Herein, we report the clinicopathological features of a case of γδ T-ALL and provide an overview of T-ALL along with recent molecular genetic findings.

## Clinical history

**Fig. 1:**
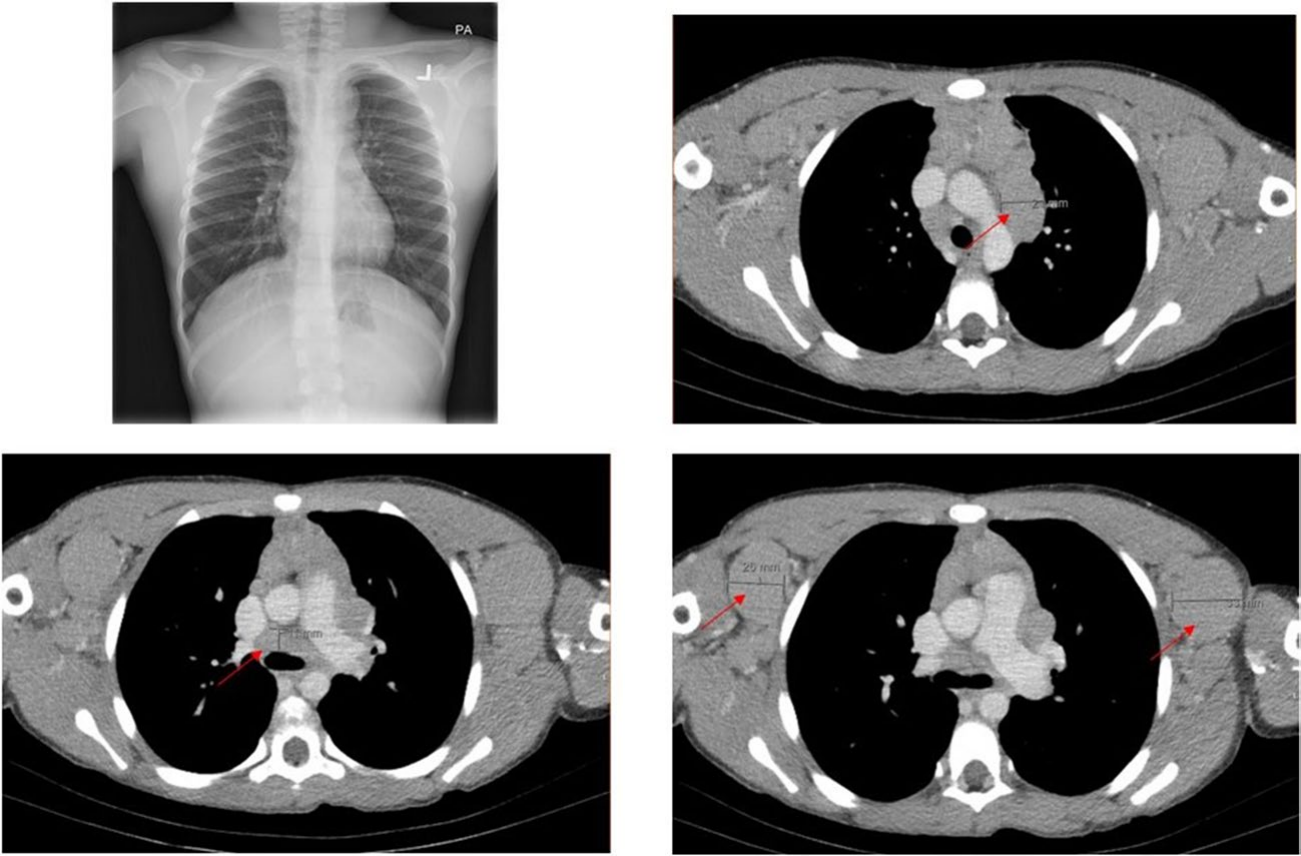
**A** Chest X-ray demonstrating right paratracheal stripe, suggesting mediastinal lymphadenopathy. Subsequent CT imaging of the chest with IV contrast demonstrated multiple enlarged lymph nodes within the **B**, **C** mediastinum and **D** bilateral axillae

An 8-year-old boy with no prior medical or surgical history presented with facial swelling, shortness of breath, and progressive cervical and axillary lymphadenopathy. He denied any constitutional symptoms including fever, night sweats, loss of weight, or appetite. A chest X-ray showed a prominent right paratracheal stripe in keeping with mediastinal lymphadenopathy (Fig. 1A). CT imaging of the chest and abdomen revealed mild splenomegaly and discrete, non-calcified, solid, non-necrotic, enlarged mediastinal, supraclavicular, axillary, intraperitoneal, and pelvic lymph nodes with associated compression of the left brachiocephalic vein and narrowing of bilateral jugular veins (Fig. 1B–D). Laboratory investigations were notable for abundant circulating blasts (75% on manual differential), in addition to an elevated lactate dehydrogenase (LDH) (414 U/L) and total leukocyte count (23,300/µL), with an increase in the absolute lymphocyte (5800/µL), monocyte (900/µL), and basophil counts (200/µL).

## Materials and methods

Tissues were fixed in 10% buffered formalin and slides of 4–6 µm sections were cut from formalin-fixed paraffin embedded tissues. Sections were stained with hematoxylin and eosin and immunohistochemistry was performed using standard procedures. Expression of cell surface markers was measured by flow cytometry (Navios, Beckman Coulter) using the ClearLLab 10C 10-color T-cell panel (Beckman Coulter) containing FITC-labeled TCR γδ antibody.

A total of 20 metaphase spreads were analyzed by Giemsa (G)-banding for chromosomal analysis. Fluorescence in situ hybridization (FISH) was performed on 200 interphase nuclei using the following ALL panel probes (Abbott Molecular/Vysis, Inc.): LSI BCR/ABL1 ES dual color translocation, LSI MLL (KMT2A) dual color, break apart rearrangement, LSI ETV6 (TEL)/ RUNX1 (AML1) ES dual color translocation, and LSI IgH dual color, break apart rearrangement probes. The following additional probes were also applied: CRLF2 break apart (Cytocell, Inc.), ABL2 break apart (Cytocell, Inc.), CEP 4 (Abbott Molecular/Vysis, Inc.), PDGFRB break apart (Cytocell, Inc.), ABL1 break apart (Cytocell, Inc.), and CEP 10 (Abbott Molecular/Vysis, Inc.) probes.

Targeted DNA-based next-generation sequencing (NGS) was performed using the 34-gene Illumina TruSeq Myeloid Panel.

## Results

**Fig. 2:**
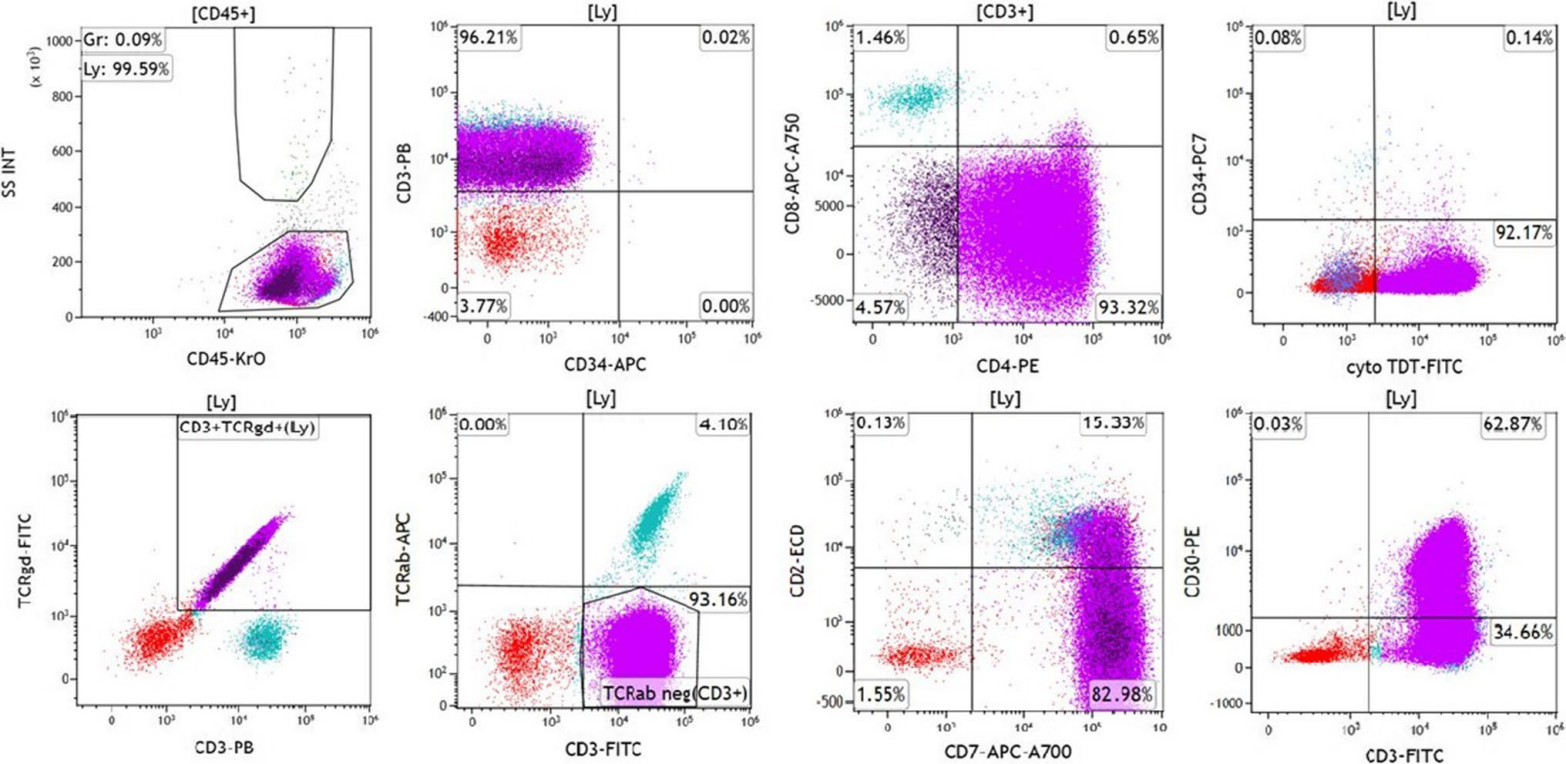
Flow cytometry evaluation of a right axillary lymph node excisional biopsy specimen revealed 91% abnormal T-cells (purple), positive for CD3, CD4, cytoplasmic terminal deoxynucleotidyl transferase (TdT), T-cell receptor (TCR) gamma delta (γδ), CD7, CD2 (partial), CD30 (subset), CD1a (not shown), CD5 (not shown), and CD38 (not shown), while negative for CD34, CD8, and other markers tested

Flow cytometry evaluation of a right axillary lymph node excisional biopsy specimen revealed 91% abnormal T-cells positive for CD4, CD3, CD2 (subset), CD5, CD7, CD38, CD1a, cytoplasmic terminal deoxynucleotidyl transferase (cyto-TdT), CD30 (subset), and TCR γδ (Fig. 2). A diagnosis of T-cell lymphoblastic lymphoma (γδ T-LBL) was rendered.

**Fig. 3:**
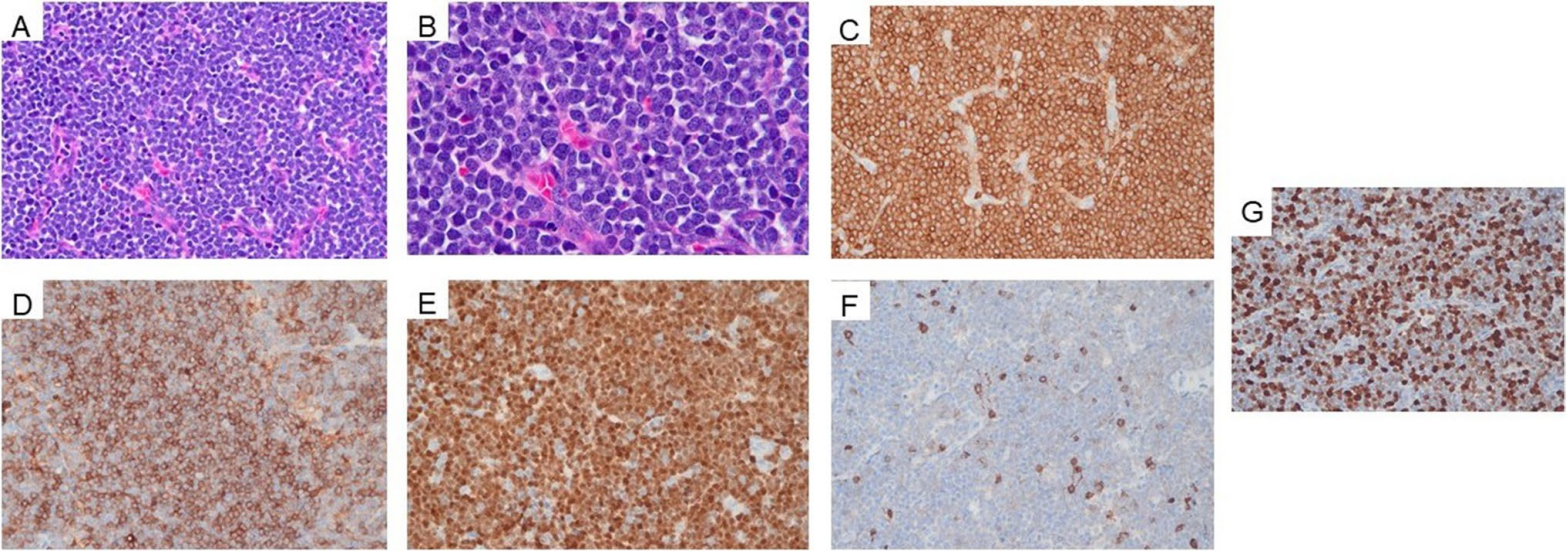
Histological examination shows serial sections of enlarged lymph node (**A** and **B**, 400 × and 500 × , respectively) involved by a dense, diffuse, neoplastic infiltrate. The neoplastic cells are positive for **C** CD3, **D** CD4, and **E** TdT, while negative for **F** CD8. **G** The Ki-67 proliferation index was noted to be high (80–90%)

Histopathological examination showed serial sections of enlarged lymph node involved by a dense, diffuse, neoplastic infiltrate with background tingible body macrophages (Fig. 3A and B). By immunohistochemistry, the neoplastic cells were positive for CD3, CD4, and TdT, while negative for CD8 (Fig. 3C–F). The Ki-67 proliferation index was high (80–90%) (Fig. 3G). Cytologically, the neoplastic cells were intermediate-to-large with a high nuclear-to-cytoplasmic ratio, coarse chromatin, and frequent mitotic figures.

Bone marrow examination showed diffuse involvement by γδ T-ALL. Chromosomal analysis revealed a normal male karyotype (46, XY) while FISH revealed a copy number loss of *PDGFRB* (5q32) in 90.5% of interphase nuclei. Targeted DNA-based NGS detected a tier II oncogenic variant in *NOTCH1* (c.7375C > T, p.Gln2459Ter) at a variant allele frequency (VAF) of 21%. The patient was started on induction chemotherapy per the AALL1231 protocol (vincristine, daunorubicin, dexrazoxane, calaspargase, and dexamethasone) and intrathecal chemotherapy with cytarabine and methotrexate. Following induction therapy, he was switched to AALL0434 and later received consolidation chemotherapy. He is presently minimal residual disease (MRD)-negative.

## Discussion

T-ALL is a T-lymphoid leukemia that accounts for nearly 15% of childhood ALL and approximately 25% adult ALL cases [4]. γδ T-ALL, in turn, accounts for only 9–12% of T-ALL [2, 3]. T-ALL is more common in adolescent males and patients typically present with a high leukocyte count, often with concurrent nodal or anterior mediastinal masses [4] The differential diagnosis in most cases includes other acute leukemias [4]. Conversely, mature γδ T-cell neoplasms include hepatosplenic T-cell lymphoma, skin and mucosal gamma delta T-cell lymphoma, and gamma-delta T-cell large granular lymphocytic leukemia (T-LGL) [4]. Microscopically, T-ALL is characterized by small to intermediate-size blasts with scant cytoplasm with occasional vacuoles, large nuclei containing condensed to dispersed chromatin, and often inconspicuous nucleoli [4]. Serial sections of lymph node in T-LBL show complete effacement of the architecture with frequent involvement of the capsule [4].

By definition, the lymphoblasts should be positive by flow cytometry for lineage-specific cytoplasmic or surface CD3 and may show variable expression of CD2, CD4, CD5, CD7, CD8, TdT, and CD1a [4]. Markers such as TdT, CD34, CD1a, CD117, and CD99 help denote the precursor nature of the blasts [4]. Aberrant expression of B-cell markers, such as CD79a, myeloid markers, such as CD13, and CD33, and NK cell markers, such as CD56, may also rarely be observed [4]. Accordingly, the immunophenotype of early T-precursor lymphoblastic leukemia (ETP-ALL) must meet all of the following diagnostic criteria: expression of cytoplasmic CD3 (with rare surface CD3 expression), absent myeloperoxidase (MPO), lack of CD1a and CD8 expression, ≥ 25% of blasts with ≥ 1 stem cell or myeloid markers (i.e., CD34, CD117, CD13, CD65, CD11b, HLA-DR), and dim-to-negative CD5 expression [4].

Cytogenetic abnormalities observed in 50–70% of T-ALL/LBL include rearrangements involving 14q11.2 (α and δ TCR loci), 7q35 (β locus), and 7p14-15 (γ locus) [4]. Other translocations include t(10;11)(p13;q14) (*PICALM::MLLT10*) and *KMT2A* (*MLL*) with *MLLT1* (*ENL* at 10p13) [4]. Clonal rearrangement of *TCR* genes is almost always seen, and clonal *IgH* rearrangement may be observed in nearly 20% of cases [4]. The most common mutations affect genes involving the *NOTCH1* pathway, *TCR* loci, epigenetic regulators (e.g., *EZH2*, *SUZ12*), chromatin modifiers (e.g., *PHF6*, *KDM6A*), *JAK-STAT* signaling pathway, *PI3K-AKT* signaling pathway, *RAS-MAPK* signaling pathway, and cell cycle regulators (e.g., *CDKN2A*, *CDKN2B*) [4].

To our knowledge, only a few cases of γδ T-ALL have been described in the literature, which all highlight the diverse phenotypic, cytogenetic, and molecular presentations [2, 3, 5–8]. For instance, Wang et al. and Kohla et al. describe cases of γδ T-cell neoplasms comprised of both mature and immature components; Kohla et al. also noted marked eosinophilia, which initially raised concern for a myeloproliferative process [2, 7]. Wei et al. and Fujino et al. both report cases of γδ T-ALL harboring unique *AF10* fusion transcripts [3, 5] Moreover, T-ALL/LBL was formerly stratified into four immunophenotypic subtypes, corresponding to stages of normal T-cell differentiation: (1) pro-T/T-I, (2) pre-T/T-II, (3) cortical T, T-III, and medullary T/T-IV [4]. Although γδ T-cells undergo thymic maturation, they do not incorporate CD4 or CD8 co-receptors and do not function through conventional MHC binding mechanisms. Interestingly, while we observed CD4 expression in our case, all reports of γδ T-ALL to date have described dim-to-absent expression of CD4 and CD8 [2, 3, 5–7]. Lastly, when compared to its αβ counterpart, γδ T-ALL was previously reported to have poorer prognosis with a CD45RA^−^/CD45RO^+^ phenotype and lower hemoglobin levels in children and splenomegaly with higher white cell counts in adults, although further studies are needed to understand the distinctions [8].

Preliminary results from a comprehensive genomic analysis based on whole-genome sequencing (WGS) and RNA-sequencing data identified several genomic subtypes of γδ T-ALL, with the *STAG2/LMO2* subtype representing a high-risk group (age at diagnosis < 3 years with poor survival outcomes) [9]. Previously, based on results from whole-exome sequencing, single nucleotide polymorphism (SNP) microarrays, and RNA transcriptome sequencing of children and young adults with newly diagnosed T-ALL enrolled on the COG trial AALL0434, 242 patients were classified into eight subgroups characterized by deregulation of *TAL1*, *TAL2*, *TLX1*, *TLX3*, *HOXA*, *LMO1*/2, *LMO2*-*LYL1*, or *NKX2*-*1* [10] Although additional comprehensive genetic studies are needed, as mutations in several signaling pathways overlap in many cases [10], γδ T-ALL requires early recognition and prompt work-up to distinguish it from ETP-ALL and mature γδ T-cell neoplasms.
